# Circadian Variation of Blood Pressure in Patients with Chronic Musculoskeletal Pain: A Cross-Sectional Study

**DOI:** 10.3390/ijerph19116481

**Published:** 2022-05-26

**Authors:** Santiago Navarro-Ledesma, Ana Gonzalez-Muñoz, Maria Carmen García Ríos, Daniel de la Serna, Leo Pruimboom

**Affiliations:** 1 Department of Physiotherapy, Faculty of Health Sciences, Campus of Melilla, University of Granada, Querol Street 5, 52004 Melilla, Spain; 2 University Chair in Clinical Psychoneuroimmunology (University of Granada and PNI Europe), 52004 Melilla, Spain; cpni.pruimboom@icloud.com; 3 Clínica Ana González, 29018 Malaga, Spain; anagonzalez.fisioterapeuta@gmail.com; 4 Department of Physiotherapy, Faculty of Health Sciences, University of Granada, 18071 Granada, Spain; mcgrios@ugr.es; 5 PNI Europe, 2518 JP The Hague, The Netherlands; danisv2001@yahoo.es; 6 Department of Physiotherapy, Faculty of Health Sciences, Universidad Pontificia de Salamanca, Gaztambide Street 12, 28015 Madrid, Spain

**Keywords:** pain, chronic pain, chronobiologic indicators, circadian rhythm disorders, blood pressure, musculoskeletal disorders

## Abstract

The aim of this study was to analyze the impact of circadian variation of blood pressure (BP) in patients with chronic musculoskeletal pain (CPM). A further purpose was to study differences in circadian variation of BP between genders and the correlation between BP circadian variation and pain. We performed a cross-sectional, observational study in which seventy-five participants with CMP participated. Circadian variation in BP was calculated using the diurnal/nocturnal BP ratio, and all participants used validated self-measurement BP devices. The Numeric Pain Rating Scale was used to assess pain perception. All circadian BP values from participants who suffered from CPM followed pathologic cardiovascular parameters (BP ratio < 10%). When comparing BP ratios between genders, statistically significant differences were found (*p* = 0.011). BP itself did not correlate with pain in any subgroup. Circadian variations of BP in those suffering from CMP are shown and new possibilities of research and treatment are proposed.

## 1. Introduction

Chronic musculoskeletal pain (CMP) is a highly prevalent condition which presents a profound impact on individuals and society, and is one of the most common forms of chronic pain [1]. Chronic musculoskeletal pain (in particular, low back pain) is the main contributor to disability worldwide [2]. Musculoskeletal pain is defined as acute or chronic pain affecting all musculoskeletal tissues including muscles, bones, ligaments, tendons, and nerves [3]. The pain and nuisance associated with musculoskeletal disorders are a common medical and socioeconomic problem worldwide [4]. CMP comprises a number of different pain syndromes, which range from local pain to neuropathic pain. CMP normally increases in daily activities, is a risk factor for drug abuse, and causes high frequency of sick leave and disability pensions, next to the negative impact on the quality of life [4].

Despite the worldwide prevalence of CMP, a complete understanding of its aetiologyand pathogenesis remains unclear and new insights are needed to help the growing group of people suffering from it. Modern life is characterized by a wide number of new risk factors with possible impacts on (long term) health and low-grade inflammation. These include increased sitting time and high caloric intake, which may be ‘accepted’ by the fast-adapting human brain but are activators of the pro-inflammatory components of the immune system [5]. Furthermore, bad sleep habits caused by the presence of artificial light and other sleep disturbing factors are important possible risk factors [6], as well as disturbances in circadian blood pressure may be, as we hypothesized, which has been shown in different neurological, inflammatory and metabolic conditions [7,8,9,10]. The circadian rhythm influences pain sensations through multiple pathways. Clock genes at medular and brain level seem to regulate the expression of several glutamate receptors related with nociception [11]. Circadian rhythm disturbances increase the expression of excitatory glutamate receptors, including NMDA, leading to long term potentiation and increased pain sensitivity [11]. Blood pressure normally follows a rhythm parallel to the circadian rhythm, with lower BP at night than in the morning, controlled by the central clock in the suprachiasmatic nucleus [12]. This decrease of the BP is called dipping and normal dipping is approximately10–20% less of the average morning blood pressure. Our hypothesis was based on the knowledge of circadian rhythm on blood pressure and its effects on pain sensations in patients suffering from CMP.

### 1.1. Epidemiology

Data from the World Health Organization (WHO) estimate that 20–33% of the world’s population has some form of chronic musculoskeletal pain, meaning that 1.75 billion people suffer globally of CMP [13]. Recent research shows that one in five adults suffer from pain each year, with a ratio of 1:10 adults with pain developing chronicity [13]. These data support the aforementioned impact of CMP not only on the health and quality of life in people suffering from CMP, but also its social and economic influence worldwide.

### 1.2. The Pathogenesis of CMP

To date, CMP is considered to be a complex puzzle whose individual pieces are not fully understood and still need to be researched. However, it is clear that different characteristics are present in this condition, such us the presence of central sensitization, the presence of a low grade inflammatory state, and its interplay with several altered brain regions involved in pain processing and behavior [1].A modern lifestyle characterized by increased sitting time, less physical activity, high caloric intake, and environmental and light pollution is highly toxic and causes the aforementioned low-grade inflammation that is related not only to CMP, but most, if not all, chronic non-communicable diseases [5].

CMP has been coined as a biopsychosocial syndrome [14]. Therefore, next to physical/biomechanical risk factors, tissue damage, sensory, emotional, cognitive, and environmental risk factors also play a role within CMP [14]. In this regard, increasing evidence suggests that artificial light pollution affecting circadian rhythms could play a vital role in health in general [9,15] and in pain especially. Prolonged disruptions to the circadian clock are associated with negative health consequences [16] as has been shown in different conditions such as neurodegenerative diseases [7] (Alzheimer’s, Parkinson’s, and Huntington’s), metabolic syndromes [8], and inflammatory diseases [9,10], including some types of cancer [9]. Artificial light and especially blue light exposure has increased dramatically in the last two decades by the use of electronic devices such as laptops, tablets, and mobile phones, and blue light affects circadian rhythm through inhibiting the production of melatonin by the epiphysis [17].

The suprachiasmatic nucleus (SCN) is the master circadian clock in mammals. The SCN serves to synchronize the timing of rhythmic activities throughout the body to the light/dark cycle. Inputs from the retino-hypothalamic tract to the SCN synchronize the endogenous clock to signals from the external environment. Studying variations of blood pressure(BP) is a way of assessing the master circadian clock, through the diurnal/nocturnal BP ratio [18].

Neurohumoral factors, which affect the autonomic nervous and cardiovascular systems, are affected when alteration of the circadian rhythm appears, and these alterations present as persistent changes in the pattern of the BP [19]. In this regard, the RAA-system alteration can disturb blood pressure and inflammation [20]. Altered melatonin levels during the night cycle can also affect immune functioning, regulation of pain sensitivity and brain functioning, as well as the production of inducible NO synthase, which may lead to endothelial dysfunction [21,22]. The same holds for the upregulating effect it has on nuclear factor erythroid 2-related factor 2 (Nrf2) [23,24].

Additionally, these alterations which are caused by a disturbed circadian rhythm could contribute to the pathogenesis of chronic disorders in general and especially to CMP, in which central sensitization is increased. This condition causes hypersensitivity to pain [25], in addition to other possibilities such as muscle injuries and peripheral injuries [26,27].

We therefore designed a cross-sectional observational study in 80 patients afflicted by CMP. The hypothesis is that the circadian blood pressure rhythm is altered in people suffering from CMP and also with the perception of pain. To our knowledge, this is the first clinical study researching a possible association between CMP, blood pressure, and an altered circadian rhythm. Circadian rhythm can be regulated by simple measurements such as the use of blue light filter glasses [28], the combination of melatonin and bright light lamps [29], and even by sleeping 2 nights in a natural environment [30]. If CMP, blood pressure, and circadian rhythm are associated, the aforementioned cheap interventions could contribute to the group of possible treatments for persons afflicted by CMP to improve quality of life, pain perception [16], and the socioeconomic impact of CMP.

The primary goal of this study is to analyze the circadian variation of BP in patients with CMP. Furthermore, to study the differences in the BP between genders and the correlation between circadian variation of BP and pain.

## 2. Methods

### 2.1. Study Design

This was a cross-sectional, observational study, in a group of 80 patients. All procedures were conducted according to the Declaration of Helsinki. Ethical approval and permission to carry out the research was obtained from the University of Granada (1948/CEIH/2021). The study has been reported following the recommendations of the STROBE statement for observational studies.

### 2.2. Sample Size

A convenience sample of 80 participants who presented with CMP, which had lasted for more than three months, was recruited from a private clinical practice in Malaga (Spain) from December 2020 to July 2021. Participants were informed of the trial through formal meetings and trial information sheets. A research assistant assessed participants for eligibility. If participants satisfied the inclusion criteria, they were included in the study. All the participants accepted and signed an informed consent before beginning the study.

Five voluntary participants were excluded from the study since they did not meet the inclusion criteria outlined in the next paragraph. Therefore, a sample comprising of 75 participants was assessed. Research assistants collected a consent form from every participant, and the confidential information collected from participants was password protected and stored.

The following additional inclusion criteria had to be met: (i) patients should be aged 18 or older, and (ii) present a primary complaint of chronic musculoskeletal pain according to the multidimensional diagnostic criteria for chronic pain [31].

Participants were ineligible to participate if any of the following conditions were present: (i) fibromyalgia, (ii) osteoarthritis, (iii) rheumatoid arthritis, (iv) spondyloarthropathies, (v) chronic gout, (vi) history of fracture; (vii) postoperative musculoskeletal pain during the previous six months; (viii) neoplastic diseases, and (ix) neurogenic pain.

*Outcome measures*

*Primary outcome measure; Diurnal/nocturnal BP ratio*

The diurnal/nocturnal BP index(BPI) is defined as the nocturnal decline in BP relative to the diurnal BP mean, and it is calculated as 100 × (mean diurnal BP − mean nocturnal BP)/mean diurnal BP. Mean BP is calculated by mean BP = DP + 1/3 (SP − DP), whereas SP stands for systolic and DP for diastolic blood pressure. Using this index, patients have been arbitrarily classified as normal dippers(diurnal/nocturnal ratio > 10%) or non-dippers (diurnal/nocturnal ratio < 10%). More recently, this classification has been extended by dividing the patients into four possible groups: extreme-dippers (diurnal/nocturnal BP ratio ≥ 20%), normal dippers (ratio ≥ 10%), non-dippers (ratio < 10%), and inverse-dippers or risers (ratio < 0%, indicating nocturnal BP above the diurnal mean) [18].

All participants were instructed to measure both diurnal (at the moment they awaken, which was between 7 a.m.–8 a.m. for all participants) and nocturnal BP (before going to bed, which was between 12 p.m. and 1 a.m. for all participants),for seven consecutive days and to send the recorded data to the research assistant who carried out the recruitment process.

Measurement procedures were based on the guidelines published by the International Society of Hypertension (ISH) and taught to the patients by the research assistants [32]. Once all the data were received, the diurnal and nocturnal BP values for the 7 days were analyzed to calculate the BP ratio for each day, and the mean of the diurnal/nocturnal ratio was calculated to be included in the data analysis. A total of 1120 BP measurements (520 diurnal/520 nocturnal) were carried out in data collection. All participants used validated self-measurement BP devices and followed the aforementioned guidelines of the ISH, producing reliable values for scientific research [33,34].

### 2.3. Secondary Outcome Measure

#### 2.3.1. Pain

The Numeric Pain Rating Scale (NPRS) was used, where 0 indicates “no pain,” and 10 indicates “worst possible pain.” Patients were asked to rate the average intensity of their pain over the past 7days. This procedure has demonstrated a high degree of validity and reliability [35].

#### 2.3.2. Data Analysis

The Statistical Package for the Social Sciences (version 23.0; SPSS Inc., Chicago, IL, USA) was used to analyze the collected data. Normality of the variables was explored using the Kolmogorov–Smirnov test. To study differences between groups, independent sample t-tests were used. A *p*-value < 0.05 was considered statistically significant. To determine the correlations between BP ratio and pain, a Spearman’s coefficient was used because of the absence of normality. Weak correlation was defined as values between 0.3 and 0.5, whereas a value of0.5 and 0.7 was considered a moderate correlation and finally strong correlation was considered greater than 0.7 [36].

## 3. Results

**Figure 1:**
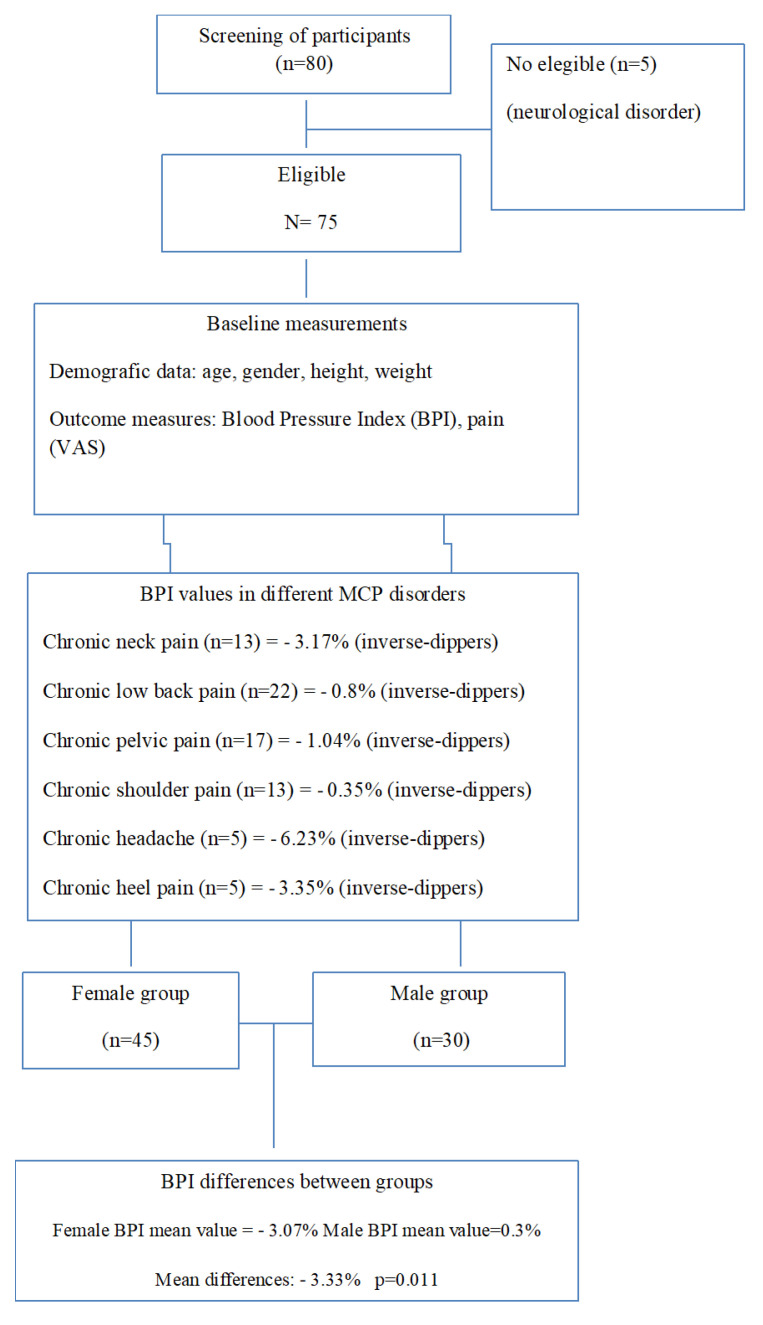
Flow diagram showing the recruitment procedures and the final number of patients included in this study.

**Figure 2:**
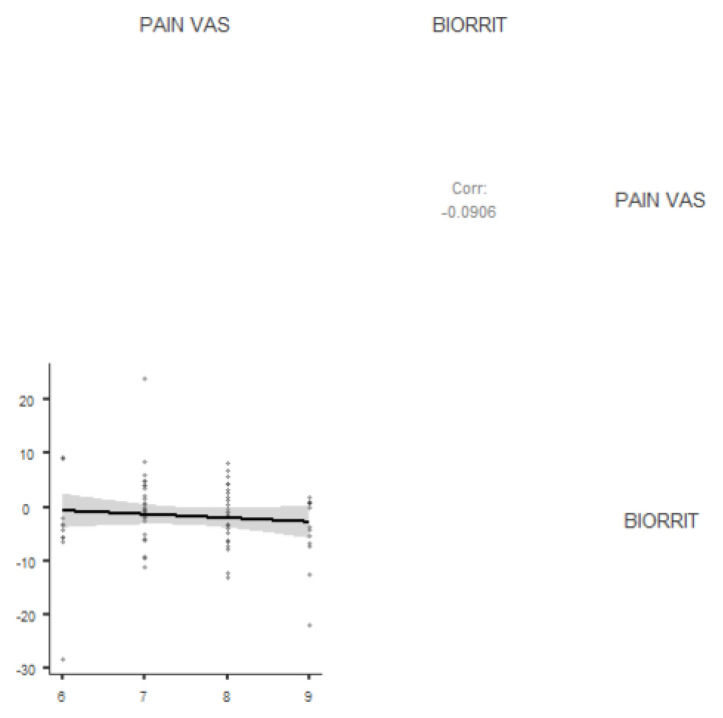
Scatter plot showing the association between BPI and pain (VAS) within the group.

A total of 80 participants were recruited, with five participants being excluded because they did not fulfill the inclusion criteria. A final number of 75 participants (45 women and 30 men) were enrolled in the study and completed the baseline assessment. The flow diagram (see Figure 1 and Figure 2) shows our recruitment procedures and the final number of patients included in this cross sectional, observational study. All participants suffered from CMP, specifically from neck pain (*n* = 13; BPI = −3.17%), low back pain (*n* = 22; BPI = −0.8%), pelvic pain (*n* = 17; BPI = −1.04%), shoulder pain (*n* = 13; BPI = −0.35%), headache (*n* = 5; BPI = −6.23%), and heel pain (*n* =−3.35; BPI = −3.35%).

Demographic characteristics and baselines of age, weight, height, BPI, and pain measures, as well as differences in BPI between genders are shown in Table 1.

The mean values of the participants were as follows: 45 years in age, 75 kg in weight, and 174 cm in height, with 45 of the participants in the study being female and 30 male.

The mean baseline BPI was −0.05 and the mean baseline pain was a score of 7. There were significant differences in weight and height between genders at baseline. No significant differences were presented in BPI or pain mean values. When comparing BP ratios between genders, statistically significant differences between groups were found (*p* = 0.011; BPI = −3.33%).

Table 2 presents the level of association between BPI and the remaining variables (pain, weight, age) as well as the level of association of pain with age and weight. There was no correlation between BPI and pain in either the female group (r = −0.124; *p* = 0.418) or the male group (r = 0.08; *p* = 0.62). In relation to BPI and weight, neither the female group (r = 0.08; *p* = 0.592) nor the male group (r = 0.103; *p* = 0.55) showed any correlation.

Finally, with regard to BPI and age, women presented no correlation (r = −0.225; *p* = 0.138) whereas a weak correlation was observed in men (r = 0.349; *p* = 0.04).

Figure 1 and Figure 2 shows the association between BPI and pain (VAS) within the group.

## 4. Discussion

The goal of the study was to analyze the circadian variation of blood pressure in patients with CMP. Other variables investigated were gender, age, and weight, and their association with pain perception. All participants suffering from CPM showed a disturbed BPI and belonged to the group of non-dippers and inverse-dippers(BPI < 10%). When comparing BPI between genders, statistically significant differences between groups were found (−3.33%, *p* = 0.011), where women showed higher nocturnal blood pressure than men (reverse dippers versus non-dippers). We did not find an association between BPI and pain perception which is in line with two studies that showed hypoalgesia with higher blood pressure in healthy controls but no pain inhibition in patients suffering from chronic pain disorders [37,38].

This study is the first analyzing circadian variation of blood pressure in patients suffering from CMP, hence comparisons with other studies are difficult. To the best of our knowledge, only two studies have analyzed the association between BP and pain perception in chronic pain, but without consideration of circadian BP changes. These studies showed no association between BP and patients with chronic low back pain, whereas a significant inverse association was noted in pain-free individuals [37,38] and these results are in line with our findings.

Coba et al. [39] found that patients in which chronic low back pain resolved over time showed a higher hypoalgesic effect of higher blood pressure than in patients in whose pain did not resolve. The analgesic effect of higher blood pressure could be explained by triggering of descending endorphin producing pathways activated by ascending activity of the C-nerve fibre [40] and this mechanism could be disturbed in patients in which chronic pain does not resolve, although BP is higher than healthy controls (36). An important study comparing pain perception and BP between healthy subjects with patients suffering from fibromyalgia syndrome (FS) confirmed that higher BP is associated with less pain (hypoalgesia) in healthy controls and this association was not found in patients suffering from FS [37]. It is known that patients suffering from FS present deficient descending inhibitory pain mechanisms and this confirms again the lack of hypoalgesia of higher blood pressure in patients in which chronic pain does not resolve [37]. We did not find any hypoalgesia effect in our participant group of higher blood pressure at night, which could reflect a disturbance in peripheral baroreflex sensitivity (36), possible small fibre damage or central sensitization [38]. In any case, in our participant group we did not find any hypoalgesia effect of higher blood pressure at night which could also mean that the hypolgesia effect that is normally observed in acute pain patients and healthy controls (36) is prevented by a disturbed circadian rhythm which is in line with studies investigating circadian rhythm with pain perception [11].

Circadian rhythm disturbances and associated BP variation are also important risk factors in many different pathologies. BP disturbances are associated with cardiovascular and renal conditions, including high risk of terminal-organ injury, particularly to the heart (left ventricular hypertrophy, CHF, and myocardial infarct), brain (stroke), and kidney (albuminuria and progression to end-stage renal failure) [18]. The majority of previous studies which analyzed BPI in the aforementioned pathologies showed participants to have non-dipper BPI values [41,42]; however, our results showed the BPI values of the group (presenting with different chronic conditions) to be even inverse-dippers (see Figure 1). These even higher blood pressure values at night, significantly more present in women than in men, did not show any hypoalgesia effect in both men and women, which means that our patient group suffering from chronic pain does not react with any pain killing mechanism caused by higher blood pressure in other populations.

As aforementioned, statistically significant differences were found (*p* = 0.01) when comparing BPI between genders, with the female participants showing values of reverse dippers (−3.07%), while male participants presented values of non-dippers (0.3%). Future research should corroborate these findings and add them to the list of differences currently found between genders, such us the immune response and organ vulnerability, the reproductive capacity, sex hormones, genetic predisposition, parental inheritance, and epigenetics [43]. This is in line with some studies which have shed light on BPI values in healthy subjects [19]. Furthermore, alternative hypotheses to address gender differences in cardiovascular events, such as differences in Ca^2+^ permeability and mitochondrial dysfunction, have also been proposed [44]. The non-dipping pattern has been reported in secondary hypertensive patients with endocrine abnormalities and autonomic nervous system dysfunction. Additionally, non-dipper patients have shown greater left ventricular hypertrophy, greater urinary albumin excretion, deterioration in renal function, and significant elevation of fibrinogen throughout the year when compared to dippers [18].

The presented results can profoundly impact on different systems which should be addressed in more detail.

Firstly, the RAA-system maintains vascular tonicity by regulating extracellular fluid volume and arterial pressure, and it is vital for survival [45]. Its alterations can disturb blood pressure, leading to chronic or acute diseases, or even sudden death [20], as well as causing other disorders such as inflammation and pain [21]. A lack of melatonin during the night cycle has multiple effects on metabolism, pain, immunology and wound healing processes, among other impacts [24,46]. The role of aldosterone, as an important activator of the NLRP3 inflammasome, has been extensively studied, as well as its participation in the increase of ROS production, which may lead to release of mitochondrial DAMPs [47]. Also, excess aldosterone is associated with cardiovascular and metabolic diseases, including endothelial dysfunction, vascular remodelling, fibrosis, and oxidative stress [21].

Secondly, sleep disturbances and a disrupted biorhythm can cause a disturbance in the functioning of different substances produced by the HPA-axis and the anti-inflammatory influence these substances have on different immune system components, which may result in immune-dysregulation and a state of low-grade inflammation. Melatonin has a profound influence on immune functioning, regulation of pain sensitivity and brain functioning [22]. Sleep disturbances and disruption of the biorhythm changes melatonin production which can lead to increased actions by inflammatory mediators such as prostaglandins and cytokines, and failure of anti-inflammatory processes due to an increase of oxidants [48]. Melatonin further downregulates the production of inducible NO synthase, inhibits the release of cyclooxygenase-2,high-mobility group box-1 signaling and toll-like receptor-4 activation, prevents the activation of the inflammasome NLRP3 and inhibits NF-κB [23,24]. All these activities decrease the possibility of prolonged inflammation and the direct/indirect consequences it causes such as chronic pain and depression. The same holds for the upregulating effect it has on nuclear factor erythroid 2-related factor 2 (Nrf2) and the increased transfer capacity of fat-derived exosomes to macrophages, further promoting M2 transformation and inhibiting fat inflammation [23,24]. Hence, the rationale behind our findings could be explained by the effects of a disturbed biorhythm, in the form of a combined disruption, on the RAA-system, the HPA-axis, and melatonin mechanisms.

### 4.1. Strengths and Weaknesses

This study presents strengths that need to be highlighted. To our knowledge, this research is the first reporting BPI values in patients suffering from CMP, and its clinical application opens new possibilities to improve both the circadian clock function and quality of life, leading to an integrated and systemic approach. Similarly, for the first time a correlation between BPI and pain perception was calculated, but showed no correlation between variables. Nevertheless, the cross-sectional design of the study and the convenience sample that participated in the study indicate the need to interpret the results with caution. The participants were not asked about the possibility and duration of exposition to blue light during the study, which may influence the results obtained. Finally, it is known that the information given by the clinical measurement of the blood pressure (BP) is limited and its values can be influenced by other factors [33]. Other lifestyle factors such as high calorie intake, a lack of physical activity, sitting time, and psycho-emotional stress could confound our findings.

### 4.2. Clinical Implications

Alteration of the circadian BP rhythm consequently affects the autonomic nervous system, neurohumoral and immune rhythms, and functioning [9]. An imbalance of sympathetic versus parasympathetic activity is the major determinant of the alterations [18]. To establish BPI monitoring as part of the clinical assessment in CMP may allow the detection of changes to prevent the development of certain pathologies, to improve an existing pathology or to improve the quality of life. Furthermore, BPI assessment may serve as an indicator of health or as an improvement after treatment programs.

Circadian misalignment has been seen to decrease wake cardiac vagal modulation by 8–15%, as determined by heart rate variability analysis, and decrease 24-h urinary epinephrine excretion rate by 7%, without a significant effect on 24-h urinary norepinephrine excretion rate. The circadian misalignment also increases 24-h serum interleukin-6, C-reactive protein, resistin, and tumor necrosis factor-α levels by 3–29%. Therefore, it seems that circadian misalignment per se increases blood pressure and inflammatory markers [10]. Furthermore, alterations in circadian rhythms alter many biological processes, such as the sleep-wake cycle, hormone secretion, cardiovascular health, glucose homeostasis, and body temperature regulation among others. These altered processes may be explained through the potential affected role of hormones such as leptin, cortisol, melatonin, and grow hormone. In this regard, the leptin plays a regulatory role in energy metabolism by increasing the activation of the sympathetic nervous system and increasing thermogenesis by increasing thyroid hormones. Thus, disruption of circadian balance can affect leptin secretion, thermogenesis, and energy homeostasis. Cortisol is a major hormone that regulates the metabolic events in the body. It increases the use of glucose, free fatty acids, and amino acids from endogenous fuel stores. High levels of cortisol function as a catabolic hormone that reduces lean body and muscle mass, and increases energy consumption. Melatonin is an important hormone in circadian synchronization. The main role of this hormone is to maintain the biological clock and to adjust the body rhythm. Synthesis and release of melatonin is stimulated in the dark, at night, while it is suppressed by light during the day. These altered sleep-wake cycle processes can also disturb the natural course of both insulin sensitivity and insulin secretion decrease at night (especially between 3:00 and 5:00 a.m.) showing the impact of circadian control on glucose metabolism. In this context, the effect of growth hormone released during the night may not be mitigated, which results in a pathologic circadian rhythm which can lead to morning hyperglycemia in dependent of eating patterns [44]. These findings may help to explain why circadian BP variations should be taken into account when assessing and treating subjects with chronic CMP.

Finally, the CNS sensitivity increase, which results from the presence of central sensitization as a consequence of an altered circadian BP rhythm may produce oxidative stress and tissue damage, thus producing Toll-like receptors which produce an activation series in the glial cell and inflammatory immune events, resulting in low-grade inflammation and increased pain perception [25].

### 4.3. Prospective

Future research with longitudinal designs which studies changes in BPI after treatments, and analyzes correlations between BPI, quality of life, and pain in time, are needed. On the other hand, research into other factors that can influence BPI (mainly those related to neurohumoral, immunological, and autonomic nervous factors and therefore cell metabolism), such us dietary habits and exercise are required [49,50,51,52]. Furthermore, building and researching biopsychosocial clinical approaches which include variations in circadian BP would strengthen and benefit the understanding and treatment of CMP.

## 5. Conclusions

This research is the first step in demonstrating circadian variations of blood pressure in those suffering from CMP. The results showed a non-dipper or inverse-dipper pattern and increased pain sensitivity, opposite to the hypoalgesia effect of higher blood pressure in healthy controls or patients with acute pain. Regulation of the biorhythm through sleep hygiene could have an important influence on the prevention of chronic pain and the prognosis for people suffering from CMP. Possible interventions to normalize the biorhythm such as the use of bright light lamps, tryptophan rich nutrients, dark room creation and regular sleep rhythm are cheap and easy to introduce into daily life. Nevertheless, further studies that address the association between a disturbed biorhythm, blood pressure and CMP are needed.

## Figures and Tables

**Table 1:** Baseline characteristics of the study group (Standard Error) and mean differences between groups.

	Female (*n* = 45)	Male (*n* = 30)	*p*	Statistical Significance	Effect Size (Cohen’s d)
Age (years)	43.9 (11.6)	47.3 (11.9)	0.004	−3.36 *p* = 0.105	−0.288
Weight (kg)	70 (13.3)	81.5 (9.19)	0.016	−12 *p* = <0.001	−0.971
Height (cm)	168 (4.64)	180 (6.75)	<0.001	−10.60 *p* = <0.001	−1.974
Blood Pressureindex (%)	−3.07% (8.31)	0.3% (3.94)	<0.001	−3.33% *p* = 0.011	−0.488
Pain (VAS 0–10)	7.42 (1.08)	7.73 (0.58)	<0.001	−3.64 *p* = 0.129	−0.341

A *p*-value < 0.05 was considered significant. VAS: Visual Analogue Scale.

**Table 2:** Correlations between variables in female and male groups.

	Female Group	Male Group
BP index and pain	r = −0.124, *p* = 0.418	r = 0.08, *p* = 0.62
BP index and weight	r = 0.08, *p* = 0.592	r = 0.103, *p* = 0.55
BP index and age	r = −0.225, *p* = 0.138	r = 0.349, *p* = 0.04 *
Pain and age	r = −0.395, *p* = 0.007 *	r = −0.205, *p* = 0.148
Pain and weight	r = 0.278, *p* = 0.065	r = 0.590, *p* = <0.001 *

* *p* < 0.05: statistically significant.

## Data Availability

Not applicable.
